# Does fine particulate matter (PM_2.5_) affect the benefits of habitual physical activity on lung function in adults: a longitudinal cohort study

**DOI:** 10.1186/s12916-020-01570-5

**Published:** 2020-05-13

**Authors:** Cui Guo, Yacong Bo, Ta-Chien Chan, Zilong Zhang, Changqing Lin, Tony Tam, Alexis K. H. Lau, Ly-yun Chang, Gerard Hoek, Xiang Qian Lao

**Affiliations:** 1 Jockey Club School of Public Health and Primary Care, The Chinese University of Hong Kong, 421, 4/F School of Public Health, Prince of Wales Hospital, Sha Tin N.T., Hong Kong SAR, China; 2 Research Center for Humanities and Social Sciences, Academia Sinica, Taipei, Taiwan; 3 Division of Environment and Sustainability, The Hong Kong University of Science and Technology, Hong Kong SAR, China; 4 Department of Civil and Environmental Engineering, The Hong Kong University of Science and Technology, Hong Kong SAR,, China; 5 Department of Sociology, The Chinese University of Hong Kong, Sha Tin N.T., Hong Kong; 6 Institute of Sociology, Academia Sinica, Taipei, Taiwan; 7 Institute for Risk Assessment Sciences, Utrecht University, Utrecht, The Netherlands; 8 Shenzhen Research Institute of the Chinese University of Hong Kong, Shenzhen, China

**Keywords:** Long-term exposure, PM_2.5_, Habitual physical activity, Lung function

## Abstract

**Background:**

Physical activity (PA) increases a person’s inhalation of air pollutants due to greater ventilation, possibly leading to larger adverse health effects. This study aims to investigate the combined effects of long-term exposure to fine particulate matter (PM_2.5_) and habitual PA on lung function in adults.

**Methods:**

This was a longitudinal cohort study that included 278,065 Taiwan residents with an age of 20 years old or above who joined a standard medical screening programme between 2001 and 2014. Each participant received at least one medical examination (including spirometric, blood, and urinary tests and a standard self-administered questionnaire survey) during the study period. We estimated the 2-year average PM_2.5_ concentrations at each participant’s address using a new physical model based on observational data. Information on the participants’ PA was collected using the standard self-administrated questionnaire. Generalised linear mixed models were used to investigate the combined effects of PM_2.5_ and PA on pulmonary function. We also performed stratified analyses by different levels of PM_2.5_ exposure and habitual PA.

**Results:**

Each 10 MET-h increase in PA was associated with a higher level of 0.20%, 0.16%, and 0.19% in forced vital capacity (FVC), forced expiratory volume in the first second (FEV_1_), and maximum mid-expiratory flow (MMEF), respectively, after adjusting for PM_2.5_ exposure and a wide range of covariates including age, sex education, body mass index, lifestyles, and health conditions. Each 10 μg/m^3^ increase in PM_2.5_ was associated with a lower FVC, FEV_1_, and MMEF (2.43%, 2.78% and 3.10%, respectively). Negative interactions were observed, and PM_2.5_ exposure was associated with a greater reduction in lung function among the participants with higher PA levels.

**Conclusions:**

We found significant negative interaction effects between long-term exposure to PM_2.5_ and habitual PA, suggesting that the increased intake of PM_2.5_ due to PA may attenuate the benefits of habitual PA on lung function. However, the PA benefits generally remained stable at different stratum of PM_2.5_ in the stratified analyses, and habitual PA may still be recommended to people residing in relatively polluted regions.

## Background

Air pollution and physical inactivity are major public health challenges worldwide. Both are closely linked to various health outcomes, including lung diseases [1], cardiovascular disease [2], and premature death [3]. The 2016 Global Burden of Disease Study shows that ambient particulate matter (PM) air pollution contributed to 4 million premature deaths globally [4]. Physical inactivity has been identified by the World Health Organization (WHO) as one of the leading risk factors for premature death [5]. It is estimated that one in four adults is not active enough. Public health campaigns promoting physical activity (PA) are increasingly used against the pandemic of physical inactivity, and WHO Member States have agreed to strive to reduce insufficient PA by 10% by 2025 [5].

PA increases the intake of air pollutants due to higher ventilation. Thus, it has become an important public health concern whether PA may be associated with larger adverse health effects by air pollution. Health guidelines are needed especially in regions with significant air pollution to inform people whether they can benefit from habitual PA. Some studies have investigated the modifying effects of short-term exposure to air pollution on the associations of PA with respiratory or cardiovascular health, but the results were inconsistent. They found that PA may mitigate [6, 7] or potentiate [8, 9] the adverse effects of air pollution, or no significant interaction effects [10, 11]. Unlike short-term exposure, the effects of long-term exposure to air pollution may not be reversible and may result in a much larger disease burden. However, information on the combined effects of long-term exposure to air pollution and habitual PA is relatively scarce. Regarding the combined effects on pulmonary health, three studies did not observe significant interaction effects between air pollution and PA on cardiopulmonary health [2, 12, 13], whilst the others reported negative [14, 15] or positive [16] interaction effects on pulmonary health. Furthermore, these studies had relatively small sample size, which might be the potential reason for the inconsistent results. We therefore conducted a longitudinal cohort study to investigate the combined effect of long-term exposure to fine particulate matter (PM_2.5_) and habitual PA on lung function in a population of 278,065 adults.

## Methods

### Study design and participants

The participants were from an ongoing longitudinal cohort in Taiwan. The cohort details have been well documented [17, 18]. Briefly, a private firm, the MJ Health Management Institution, has provided a medical screening programme that Taiwanese residents can join with a paid membership since 1994. This programme includes a series of standard medical examinations such as anthropometric measurements and physical examinations (including spirometric, blood, and urinary tests). A standard self-administered questionnaire is used in each medical examination to collect demographic and socioeconomic information, lifestyle indicators, and medical history. The participants are encouraged to undergo annual medical examinations. Data generated from the medical examinations have been stored electronically since 1996. More than 0.5 million participants were recruited between 1996 and 2014. Each participant was required to give written informed consent before participation. The Joint Chinese University of Hong Kong - New Territories East Cluster Clinical Research Ethics Committee approved this study.

Figure S1 (Additional file: The flow chart of participants selection) shows our procedure for participant selection. We selected 342,626 participants at least 20 years of age with spirometric measurements taken during 2001–2014, when the ground-level concentration of PM_2.5_ exposure was available. Of these, 64,561 participants were excluded because of incomplete information (2668 for PM_2.5_ concentration due to missing address, 35,985 for other covariates, and 25,908 for FEV_1_/FVC ≥ 100% possibly due to technical error). We finally included 278,065 participants with 567,557 observations, of which 115,959 (41.7%) had more than one medical visits.

### PM_2.5_ exposure assessment

The details of the assessment have been described elsewhere [17, 19, 20]. In brief, we derived the aerosol optical depth (AOD) at a resolution of 1 × 1 km^2^ from the Moderate Resolution Imaging Spectroradiometer (MODIS) instruments, which were aboard the Terra and Aqua satellites launched by US National Aeronautics and Space Administration. We developed a new physical model based on observational data using the retrieved AOD data to estimate the ground-level concentration of PM_2.5_ [19]. We validated the model by comparing the satellite-based estimates with concentrations from more than 70 monitoring stations in Taiwan. The corresponding correlation coefficients ranged from 0.72 to 0.83 [17].

The address of each participant was noted during each medical visit so that the medical report could be mailed to them. Thus, any change of address was recorded. The participants’ addresses were geocoded into latitude and longitude, and we matched the addresses with the estimated PM_2.5_ concentrations. We calculated the annual average PM_2.5_ concentrations for the calendar year of the medical examination and for the previous year. We used the mean of these two averages (2-year average) as an indicator of long-term exposure to ambient PM_2.5_ air pollution.

### Habitual PA

The details of habitual PA have been described elsewhere [18, 21, 22]. In brief, a self-administered questionnaire was used in each medical examination to collect information on habitual PA. The participants’ weekly leisure activity, conducted in the month before their medical examination, was classified into four intensity categories by asking the question “Which types of physical activities did you usually take in the previous month?” with several examples given under each category: light (e.g. walking), moderate (e.g. climbing), medium-vigorous (e.g. jogging), or high-vigorous (e.g. running). These four intensity categories were assigned metabolic equivalent values (MET; 1 MET = 1 kcal/h/kg of bodyweight) of 2.5, 4.5, 6.5, and 8.5, respectively [18, 23]. The weekly total time spent on the PA was obtained by asking the question “How many hours did you spend on the PA weekly in the previous month” before 2009. Since 2009, we had used the two assessment questions to obtain the weekly total time by asking “How often did you usually do the PA weekly in previous month” and “How many hours did you spend on the PA each time”. The weekly total time spent on PA was calculated by multiplying the hours and frequency.

A weighted MET was assigned to participants who reported activities more than one intensity category, depending on the time spent in each category. The volume of activity (MET-h) was then calculated using the product of intensity (MET) and duration (hours) per week. On the basis of PA guidelines for Americans and previous studies [18, 24], the participants were further grouped into four categories based on their MET-h value: inactive PA (< 3.75 MET-h), low PA (3.75–7.49 MET-h), moderate PA (7.50–16.49 MET-h), or high PA (≥ 16.50 MET-h).

### Outcome measurements

We used the following three lung function parameters as the health outcomes: forced vital capacity (FVC), forced expiratory volume in the first second (FEV_1_), and maximum mid-expiratory flow (MMEF). The details of the spirometry test have been described in our previous publication [17, 25]. Trained professionals performed the pre-bronchodilator spirometric tests. The procedures strictly followed the guideline of the American Thoracic Society [26]. All participants were required to blow at least three times in a standing position using the MICROSPIRO HI-501 (Fällanden, Switzerland) or CHESTGRAH HI-701 (Chest M.I., Tokyo, Japan). These two spirometer models were comparable. An old spirometer was replaced with a new one if the old one was broken or regarded as abnormal because of the issues of accuracy and operation, etc. At least two blows had to be reproducible within a 5% margin for both FVC and FEV_1_. The FVC and FEV_1_ were derived from the curve with the largest values of FVC and/or FEV_1_. The MMEF measurement was from the curve with the largest sum of FVC and FEV_1_. For quality control, it was mandatory to conduct calibration check every day and document any changes to the computer and/or software and equipment repair or relocation [26].

### Covariates

Our previous publications and technical reports of MJ Health Research Foundation have provided detailed information on health measurement and quality control [17, 27]. Participant’s height and weight were measured with light clothing but no shoes. Information on demographic and lifestyle factors, and medical history was collected using a standard self-administrated questionnaire. In addition to habitual PA, the participants were asked to categorise their physical labour intensity at work according to different levels of exertion: mostly sedentary (e.g. clerk), sedentary with occasional walking (e.g. seamstress), mostly standing or walking (e.g. retail salesperson), or hard labour (e.g. porter). The variable “physical labour intensity at work” was taken into account as a covariate because this study focused on ambient PM_2.5_ and leisure-habitual PA.

In this study, the following covariates were used for the data analysis: age (years), sex (male or female), education [high school or lower (≤ 12 years), college or university (13–16 years), or postgraduate (> 16 years)], body mass index [BMI, calculated as weight divided by height squared (kg/m^2^)], smoking status (never, former, or current), alcohol drinking (never/seldom, former, or current), physical labour at work (mostly sedentary, sedentary with occasional walking, mostly standing or walking, or hard labour), vegetable and fruit intake [seldom (< 1 serving/day), moderate (1–2 servings/day), or frequent (> 2 servings/day)], occupational exposure (dust or solvent: yes or no), hypertension (defined as systolic blood pressure ≥ 140 mmHg, diastolic blood pressure ≥ 90 mmHg, or self-reported hypertension), diabetes (defined as fasting blood glucose ≥ 126 mg/dl or self-reported physician-diagnosed diabetes), dyslipidaemia (defined as total cholesterol ≥ 240 mg/dl, triglyceride ≥ 200 mg/dl, or HDL-C < 40 mg/dl), self-reported physician-diagnosed cardiovascular disease (yes or no), self-reported physician-diagnosed cancer (yes or no), and calendar year and season (spring: March to May; summer: June to August; autumn: September to November; or winter: December to February).

### Statistical analysis

We conducted the longitudinal data analysis using the generalised linear mixed model (GLMM). The three lung function parameters were logarithmically transformed to normalise the data for the analysis. The aforementioned covariates were gradually included into the following two models to control for their potential effects: crude model did not adjust for any covariates; model 1 adjusted for age, sex, BMI, education, season, and calendar year; model 2 further adjusted for smoking, drinking, vegetable intake, fruit intake, occupational exposure, physical labour at work, hypertension, diabetes, dyslipidaemia, and self-reported cardiovascular disease and cancer. The results are shown using the percentage difference in lung function for each 10 μg/m^3^ increase of PM_2.5_ or with the first quartile of the PM_2.5_ as the reference and for every 10 MET-h increase in PA or with inactive PA as the reference.

To investigate the main effects on lung function, PM_2.5_ exposure and PA were analysed separately using the two above-mentioned models. We also estimated the adjusted main effects by mutually controlling for both PM_2.5_ exposure and habitual PA in model 2. To check the interaction effect of PM_2.5_ and PA, we further included an overall interaction term between incremental PM_2.5_ (every 10 μg/m^3^) and incremental PA (every 10 MET-h). We also conducted subgroup analyses stratified by PM_2.5_ quartiles and PA categories.

We investigated the combined effects by classifying the participants into 16 groups according to PM_2.5_ quartiles and PA categories, with the reference to inactive participants with exposure to the 4th quartile of PM_2.5_. We further compared the effects of different PA levels at the lowest and highest 10% of PM_2.5_ exposure.

A series of sensitivity analyses were performed to test the robustness of the combined effects by (1) conducting a cross-sectional analysis based only on the baseline data (the first medical examination) using a generalised linear model to examine whether the combined effects were different from longitudinal data analysis; (2) excluding participants who had only one medical examination, to investigate whether the combined effects were different from the main analysis; (3) excluding participants with previous diagnoses of cancer, asthma, and chronic obstructive pulmonary disease (COPD) to eliminate the potential effects of comorbidities, including reverse causation bias (i.e. people with serious lung function impairment or diseases which blocked them from doing PA); (4) excluding participants aged < 25 years old to eliminate the potential effects of lung function growth in their early twenties; (5) excluding participants who provided a company address to control for misclassification of exposure due to different address types; (6) excluding participants who were ever smokers to control for the potential modifying effects of smoking; and (7) conducting two more analyses based on different combinations of covariates included in the model (i.e. model 1 covariates plus lifestyles and PA, and model 1 covariates plus lifestyles, occupational exposures, and PA) to examine whether lifestyles and occupational exposure modified the combined associations differently.

We conducted the statistical analyses using R 3.3.2. (R Core Team, Vienna, Austria). The estimated effects were interpreted as statistically significant with the two-tailed *P* value < 0.05.

## Results

Table 1 presents the general characteristics of the participants at baseline (the first medical examination) and all observations. The baseline data shows that participants were generally well educated. Most had never smoked and did not consume alcohol. Regarding physical labour at work, more than half were mostly sedentary. Approximately half (49.4%) of the participants were physically inactive. Compared to the baseline, a larger mean of age was observed for all observations. We also observed lower percentages of physical inactivity and seldom intake of vegetable and fruit. The number of medical visits ranged from 2 to 21 with a mean of 3.5. The median visit interval was 18 months [interquartile range (IQR) 13–30]. Compared with the participants included in the analysis, the excluded 64,561 participants had similar distributions in age (mean 45.2 vs. 43.1 years), sex (male 43.1% vs. 51.3%), education (lower than high school 23.5% vs. 15.7%), smoking status (never 77.5% vs. 74.6%), habitual PA (mean 8.4 vs. 8.4 MET-h), and PM_2.5_ concentration (mean 26.3 vs. 26.6 μg/m^3^).

**Table 1:** Characteristics of the participants

Characteristics	Participants at baseline^a^ (*n* = 278,065)	All observations^b^ (*n* = 567,557)
Age, years	40.9 (13.0)	43.1 (12.6)
Male, *n* (%)	138,499 (49.8)	291,103 (51.3)
Education, *n* (%)		
Lower than high school	48,922 (17.6)	89,013 (15.7)
High school	57,474 (20.7)	114,118 (20.1)
College or university	140,069 (50.4)	292,624 (51.6)
Postgraduate	31,600 (11.4)	71,802 (12.7)
Smoking status, *n* (%)		
Never	203,559 (73.2)	423,491 (74.6)
Former	16,459 (5.9)	35,416 (6.2)
Current	58,047 (20.9)	108,650 (19.1)
Alcohol consumption, *n* (%)		
Never	236,829 (85.2)	481,559 (84.8)
Former	26,887 (9.7)	56,962 (10.0)
Current	14,349 (5.2)	29,036 (5.1)
Physical labour at work, *n* (%)		
Mostly sedentary	172,458 (62.0)	370,365 (65.3)
Sedentary with occasional walking	74,984 (27.0)	143,804 (25.3)
Mostly standing or walking	24,448 (8.8)	43,375 (7.6)
Hard labour	6175 (2.2)	10,013 (1.8)
Physical activity (PA)		
Category, *n* (%)		
Inactive	137,261 (49.4)	252,724 (44.5)
Low	56,196 (20.2)	115,971 (20.4)
Medium	47,910 (17.2)	110,636 (19.5)
High	36,698 (13.2)	88,226 (15.5)
Continuous (kcal/kg/h)		
MET-h, median (IQR)	3.8 (9.8)	3.8 (10.9)
Vegetable intake, *n* (%)		
Seldom	38,671 (13.9)	66,845 (11.8)
Moderate	164,130 (59.0)	332,228 (58.5)
Frequent	75,264 (27.1)	168,484 (29.7)
Fruit intake, *n* (%)		
Seldom	91,781 (33.0)	160,836 (28.3)
Moderate	151,789 (54.6)	324,741 (57.2)
Frequent	34,495 (12.4)	81,980 (14.4)
Occupational exposure (solvent/dust), *n* (%)	22,978 (8.3)	44,180 (7.8)
Body mass index, kg/m^2^	23.2 (3.7)	23.3 (3.6)
Hypertension, *n* (%)^c^	46,614 (16.8)	97,116 (17.1)
Diabetes, *n* (%) ^d^	14,041 (5.0)	30,166 (5.3)
Cardiovascular disease, *n* (%)	8912 (3.2)	19,505 (3.4)
Dyslipidaemia, *n* (%)^e^	69,343 (24.9)	142,123 (25.0)
Cancer, *n* (%)	3500 (1.3)	8909 (1.6)
FVC (l)	2.9 (1.2)	2.9 (1.2)
FEV_1_ (l)	2.6 (1.1)	2.6 (1.1)
MMEF (l/s)	3.3 (1.5)	3.4 (1.5)
PM_2.5_ (μg/m^3^)^f^	26.7 (7.8)	26.6 (7.5)
PM_2.5_ by PA categories		
Inactive	26.7 (7.7)	26.6 (7.5)
Low	26.8 (7.7)	26.7 (7.5)
Medium	26.9 (7.8)	26.8 (7.6)
High	26.5 (7.8)	26.4 (7.5)

The statistics are shown as mean (standard deviation) for continuous variables, count (percentage) for categorical variables and median (interquartile range) for physical activity

*IQR* interquartile range, *FVC* forced vital capacity, *FEV*_*1*_ forced expiratory volume in 1 s, *MMEF* maximum mid-expiratory flow

^a^Characteristics of the 278,065 participants at baseline

^b^Characteristics of the 567,557 observations from the 278,065 participants

^c^Hypertension: systolic blood pressure ≥ 140 mmHg, diastolic blood pressure ≥ 90 mmHg, or reported physician-diagnosed hypertension

^d^Diabetes: fasting blood glucose ≥ 126 mg/dl or reported physician-diagnosed diabetes

^e^Dyslipidaemia: total cholesterol ≥ 240 mg/dl, triglyceride ≥ 200 mg/dl, or high-density lipoprotein cholesterol < 40 mg/dl

^f^The average PM_2.5_ levels of the year of the visit and the year before the visit

**Fig. 1:**
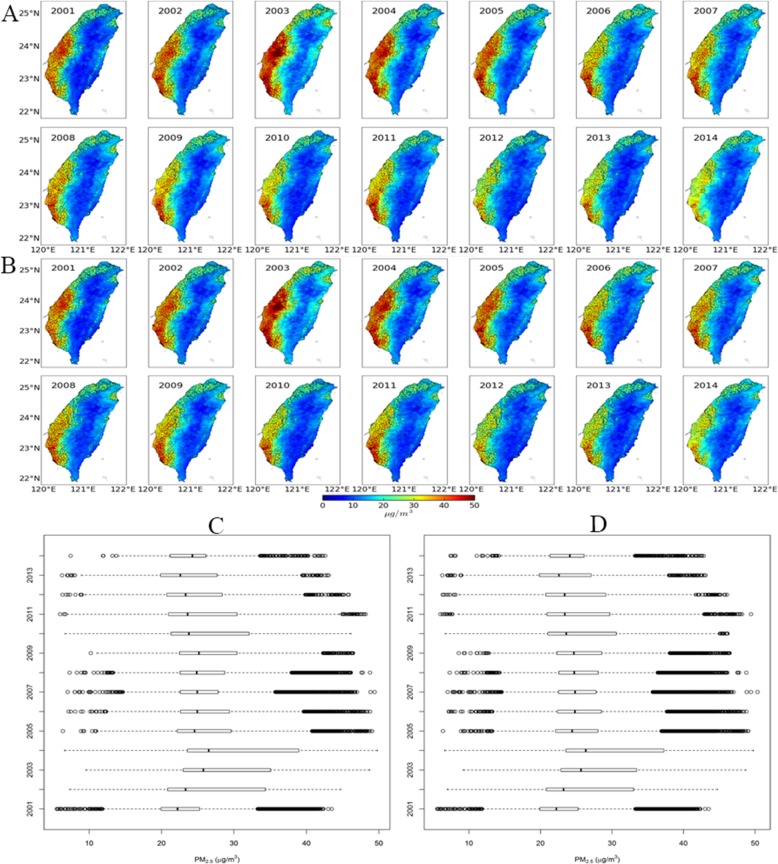
Locations of the participants and the temporal and spatial distributions of PM_2.5_ concentrations in Taiwan. **a**, **b** Sketch maps showing the locations of the participants and the spatial distribution of PM_2.5_ by year. The address locations of the participants (**a**) and their observations (**b**) were represented by circles. Circles were highly overlapped because of the large sample sizes. **c**, **d** Distributions of the PM_2.5_ concentrations by year. The centre line is the median concentration, and the tow heads of the box indicate the 25–75th percentile (IQR). Whiskers show the observations within 3 IQR, and other extreme observations are circles. **a**, **c** Distributions of 278,065 participants at baseline. **b**, **d** Distributions of the 567,557 observations from the 278,065 participants

Figure 1 shows the spatial and temporal distribution of the 2-year average of PM_2.5_ concentrations by year. Most participants lived in Western areas of Taiwan. PM_2.5_ concentrations were higher in south-western areas. There were large spatial contrasts in PM_2.5_ exposure. The spatial distribution of the PM_2.5_ concentrations was generally stable during the study period. The overall mean was 26.7 μg/m^3^ [standard deviation (SD) 7.8] for 278,065 participants and 26.6 (SD 7.5) for 567,557 observations (Table 1). The averages of PM_2.5_ exposure were generally similar in different PA categories (Table 1).

Table 2 presents the associations of lung function with PA and PM_2.5_ exposure. PA was positively associated with lung function, whilst negative associations were observed for lung function and PM_2.5_. Adjustment for a wide range of covariates yielded similar results. Interaction tests (the last column in Table 2) show that PM_2.5_ exposure was significantly more harmful to participants who undertook high PA.

**Table 2:** Associations of lung function with habitual physical activity and PM_2.5_ exposure in Taiwanese adults

	Crude model^a^		Adjusted model 1^a^		Adjusted model 2^a^		PM_2.5_ and PA together^b^		PM_2.5_*PA	
	% difference	*P*	% difference	*P*	% difference	*P*	% difference	*P*	% difference	*P*
**FVC**									− 0.13 (− 0.16, − 0.09)^c^	< 0.001^c^
Low PA	− 0.33 (− 0.42, − 0.24)	< 0.001	0.32 (0.23, 0.40)	< 0.001	0.28 (0.19, 0.36)	< 0.001	0.04 (− 0.05, 0.12)	0.370	0.10 (− 0.01, 0.22)	0.075
Moderate PA	0.00 (− 0.10, 0.10)	0.974	0.70 (0.60, 0.79)	< 0.001	0.55 (0.46, 0.65)	< 0.001	0.43 (0.34, 0.52)	< 0.001	− 0.12 (− 0.24, 0.01)	0.063
High PA	1.22 (1.11, 1.33)	< 0.001	1.19 (1.08, 1.29)	< 0.001	1.30 (1.19, 1.41)	< 0.001	0.88 (0.77, 0.99)	< 0.001	− 0.23 (− 0.37, − 0.09)	0.001
Per 10 MET-h	0.39 (0.36, 0.41)	< 0.001	0.22 (0.19, 0.24)	< 0.001	0.26 (0.23, 0.28)	< 0.001	0.20 (0.17, 0.22)	< 0.001	–	–
**PM**_**2.5**_										
2nd quartile	− 1.78 (− 1.88, − 1.68)	< 0.001	− 1.41 (− 1.50, − 1.32)	< 0.001	− 1.37 (− 1.46, − 1.27)	< 0.001	− 1.22 (− 1.32, − 1.13)	< 0.001	–	–
3rd quartile	− 2.79 (− 2.91, − 2.67)	< 0.001	− 2.19 (− 2.30, − 2.07)	< 0.001	− 2.25 (− 2.36, − 2.14)	< 0.001	− 1.94 (− 2.05, − 1.82)	< 0.001	–	–
4th quartile	− 4.98 (− 5.16, − 4.80)	< 0.001	− 4.43 (− 4.58, − 4.28)	< 0.001		< 0.001	− 3.92 (− 4.07, − 3.77)	< 0.001	–	–
Per 10 μg/m^3^	− 3.11 (− 3.21, − 3.02)	< 0.001	− 2.17 (− 2.24, − 2.09)	< 0.001	− 2.59 (− 2.67, − 2.52)	< 0.001	− 2.43 (− 2.51, − 2.36)	< 0.001	–	–
**FEV**_**1**_									− 0.21 (− 0.25, − 0.18) ^c^	< 0.001^c^
Low PA	− 0.43 (− 0.52, − 0.34)	< 0.001	0.05 (0.04, 0.06)	< 0.001	− 0.09 (− 0.18, 0.01)	0.078	− 0.09 (− 0.09, − 0.08)	< 0.001	− 0.01 (− 0.14, 0.12)	0.879
Moderate PA	0.06 (− 0.03, 0.16)	0.206	0.42 (0.41, 0.43)	< 0.001	0.48 (0.38, 0.59)	< 0.001	0.32 (0.31, 0.33)	< 0.001	− 0.15 (− 0.29, − 0.01)	0.030
High PA	1.57 (1.46, 1.68)	< 0.001	1.21 (1.20, 1.22)	< 0.001	1.11 (0.99, 1.24)	< 0.001	1.16 (1.15, 1.17)	< 0.001	− 0.43 (− 0.58, − 0.27)	< 0.001
Per 10 MET-h	0.49 (0.47, 0.52)	< 0.001	0.28 (0.25, 0.30)	< 0.001	0.23 (0.22, 0.24)	< 0.001	0.16 (0.15, 0.16)	< 0.001	–	–
**PM**_**2.5**_										
2nd quartile	− 2.59 (− 2.69, − 2.50)	< 0.001	− 2.02 (− 2.03, − 2.01)	< 0.001	− 1.66 (− 1.76, − 1.55)	< 0.001	− 1.89 (− 1.89, − 1.88)	< 0.001	–	–
3rd quartile	− 3.96 (− 4.08, − 3.84)	< 0.001	− 3.17 (− 3.18, − 3.17)	< 0.001	− 2.47 (− 2.59, − 2.35)	< 0.001	− 2.61 (− 2.62, − 2.60)	< 0.001	–	–
4th quartile	− 6.29 (− 6.47, − 6.11)	< 0.001	− 5.70 (− 5.71, − 5.69)	< 0.001	− 4.92 (− 5.06, − 4.78)	< 0.001	− 4.92 (− 4.93, − 4.91)	< 0.001	–	–
Per 10 μg/m^3^	− 4.00 (− 4.09, − 3.90)	< 0.001	− 2.67 (− 2.68, − 2.66)	< 0.001	− 2.90 (− 2.91, − 2.89)	< 0.001	− 2.78 (− 2.79, − 2.77)	< 0.001	–	–
**MMEF**									− 0.47 (− 0.52, − 0.41) ^c^	< 0.001^c^
Low PA	− 0.51 (− 0.65, − 0.37)	< 0.001	0.20 (0.06, 0.34)	0.004	− 0.23 (− 0.36, − 0.09)	0.001	0.19 (0.05, 0.33)	0.006	0.45 (0.26, 0.63)	< 0.001
Moderate PA	− 0.01 (− 0.17, 0.14)	0.889	0.29 (0.14, 0.44)	< 0.001	0.12 (− 0.03, 0.27)	0.106	0.33 (0.19, 0.48)	< 0.001	− 0.07 (− 0.27, 0.13)	0.488
High PA	2.36 (2.18, 2.54)	< 0.001	1.13 (0.96, 1.31)	< 0.001	1.14 (0.97, 1.32)	< 0.001	1.00 (0.83, 1.18)	< 0.001	− 1.47 (− 1.69, − 1.24)	< 0.001
Per 10 MET-h	0.77 (0.73, 0.81)	< 0.001	0.19 (0.15, 0.23)	< 0.001	0.23 (0.19, 0.27)	< 0.001	0.19 (0.15, 0.23)	< 0.001	–	–
**PM**_**2.5**_										
2nd quartile	− 4.38 (− 4.53, − 4.22)	< 0.001	− 3.53 (− 3.68, − 3.39)	< 0.001	− 3.67 (− 3.82, − 3.53)	< 0.001	− 3.28 (− 3.42, − 3.13)	< 0.001	–	–
3rd quartile	− 6.76 (− 6.94, − 6.58)	< 0.001	− 5.47 (− 5.64, − 5.30)	< 0.001	− 5.46 (− 5.63, − 5.29)	< 0.001	− 4.96 (− 5.13, − 4.78)	< 0.001	–	–
4th quartile	− 9.12 (− 9.36, − 8.87)	< 0.001	− 6.80 (− 7.02, − 6.58)	< 0.001	− 6.62 (− 6.85, − 6.40)	< 0.001	− 6.63 (− 6.85, − 6.41)	< 0.001	–	–
Per 10 μg/m^3^	− 5.24 (− 5.37, − 5.11)	< 0.001	− 3.18 (− 3.30, − 3.07)	< 0.001	− 3.25 (− 3.37, − 3.14)	< 0.001	− 3.10 (− 3.22, − 2.99)	< 0.001	–	–

Lung function was logarithmically transformed to normalise the data for analysis, and then the original scale was transformed back to present the effects as percentage (%) difference in lung function parameters with 95% confidence interval. The effects were presented as % difference in lung function with 95% confidence level

Participants who were in inactive PA category or in the 1^st^ quartile of PM_2.5_ comprised the reference group

The 1^st^, 2^nd^, 3^rd^, and 4^th^ quartile of PM_2.5_ was < 21.67, 21.67–24.14, 24.14–28.81, and ≥ 28.81 μg/m^3^, respectively

*PA* physical activity, *FVC* forced vital capacity, *FEV*_*1*_ forced expiratory volume in 1 s, *MMEF* maximum mid-expiratory flow

^a^Crude model: no adjustment; model 1: adjusted for age, sex, educational level, body mass index, season, and calendar year; adjusted model 2: further adjusted for physical labour at work, smoking, drinking, vegetable intake, fruit intake, occupational exposure, hypertension, diabetes, hyperlipidaemia, self-reported cardiovascular disease, and self-reported cancer

^b^Both PM_2.5_ and PA were introduced into fully adjusted models together to adjust for each other

^c^*P* value and % difference in lung function for the overall interaction term of “PM_2.5_ (10 μg/m^3^) and PA categories (inactive PA, low PA, moderate PA, or high PA)”

Positive associations between PA and lung function were also observed in stratified analyses by PM_2.5_ quartiles (Table 3). However, exposure to PM_2.5_ was associated with reduced lung function in stratified analyses by PA categories (Table 3). The reduction values for each 10 μg/m^3^ increase in PM_2.5_ were slightly larger in the participants who undertook high PA than those with lower levels of PA.

**Table 3:** Results of stratified analyses by habitual physical activity and PM2.5 quartiles in Taiwanese adults

**Stratified by PM**_**2.5**_	**1st quartile**		**2nd quartile**		**3rd quartile**		**4th quartile**	
	**% difference**	***P***	**% difference**	***P***	**% difference**	***P***	**% difference**	***P***
**FVC**								
Low PA	0.14 (− 0.08, 0.35)	0.209	0.09 (− 0.09, 0.26)	0.326	0.17 (0.01, 0.33)	0.036	− 0.20 (− 0.35, − 0.05)	0.009
Moderate PA	0.49 (0.26, 0.72)	< 0.001	0.43 (0.23, 0.62)	< 0.001	0.45 (0.27, 0.63)	< 0.001	0.14 (− 0.02, 0.31)	0.088
High PA	0.82 (0.57, 1.07)	< 0.001	1.23 (1.01, 1.46)	< 0.001	0.87 (0.65, 1.09)	< 0.001	0.57 (0.37, 0.76)	< 0.001
Per 10 MET-h	0.14 (0.08, 0.19)	< 0.001	0.23 (0.17, 0.28)	< 0.001	0.20 (0.15, 0.25)	< 0.001	0.20 (0.15, 0.24)	< 0.001
**FEV**_**1**_								
Low PA	0.03 (− 0.16, 0.22)	0.766	0.23 (0.06, 0.40)	0.010	−0.14 (− 0.29, 0.02)	0.078	− 0.06 (− 0.22, 0.09)	0.416
Moderate PA	0.43 (0.22, 0.63)	< 0.001	0.33 (0.14, 0.52)	0.001	0.13 (−0.04, 0.31)	0.136	0.44 (0.27, 0.60)	< 0.001
High PA	0.93 (0.70, 1.16)	< 0.001	1.13 (0.90, 1.35)	< 0.001	0.51 (0.30, 0.72)	< 0.001	1.10 (0.90, 1.30)	< 0.001
Per 10 MET-h	0.22 (0.17, 0.27)	< 0.001	0.18 (0.13, 0.23)	< 0.001	0.14 (0.09, 0.19)	< 0.001	0.20 (0.19, 0.22)	< 0.001
**MMEF**								
Low PA	− 0.49 (− 0.78, − 0.2)	0.001	0.26 (− 0.01, 0.54)	0.061	0.13 (−0.13, 0.39)	0.320	−0.07 (− 0.33, 0.19)	0.581
Moderate PA	0.20 (− 0.11, 0.52)	0.202	0.08 (− 0.22, 0.39)	0.594	0.29 (0.00, 0.58)	0.048	0.21 (−0.07, 0.50)	0.140
High PA	1.04 (0.70, 1.40)	< 0.001	1.00 (0.65, 1.36)	< 0.001	0.33 (−0.01, 0.68)	0.060	0.76 (0.43, 1.09)	< 0.001
Per 10 MET-h	0.31 (0.23, 0.39)	< 0.001	0.22 (0.14, 0.30)	< 0.001	0.10 (0.01, 0.18)	0.021	0.13 (0.05, 0.20)	0.001
**Stratified by PA**	**Inactive PA**		**Low PA**		**Moderate PA**		**High PA**	
	**% difference**	***P***	**% difference**	***P***	**% difference**	***P***	**% difference**	***P***
**FVC**								
2nd quartile	− 1.59 (− 1.73, − 1.45)	< 0.001	− 0.86 (− 1.06, − 0.65)	< 0.001	− 1.28 (− 1.50, − 1.06)	< 0.001	− 1.13 (− 1.16, − 1.11)	< 0.001
3rd quartile	− 2.32 (− 2.48, − 2.15)	< 0.001	− 1.20 (− 1.44, − 0.96)	< 0.001	− 1.84 (− 2.10, − 1.58)	< 0.001	− 2.62 (− 2.64, − 2.60)	< 0.001
4th quartile	− 4.96 (− 5.17, − 4.75)	< 0.001	− 4.14 (− 4.44, − 3.84)	< 0.001	− 4.40 (− 4.72, − 4.07)	< 0.001	− 4.55 (− 4.57, − 4.52)	< 0.001
Per 10 μg/m^3^	− 2.20 (− 2.31, − 2.09)	< 0.001	− 1.97 (− 2.12, − 1.82)	< 0.001	−2.48 (− 2.50, − 2.47)	< 0.001	− 3.07 (− 3.26, − 2.87)	< 0.001
**FEV**_**1**_								
2nd quartile	− 1.81 (− 1.96, − 1.67)	< 0.001	− 1.16 (− 1.36, − 0.95)	< 0.001	− 1.82 (− 2.04, − 1.59)	< 0.001	− 2.52 (− 2.77, − 2.27)	< 0.001
3rd quartile	− 2.63 (− 2.80, − 2.46)	< 0.001	− 1.90 (− 2.14, − 1.67)	< 0.001	−2.75 (− 3.01, − 2.49)	< 0.001	− 3.78 (− 4.08, − 3.48)	< 0.001
4th quartile	− 5.05 (− 5.27, − 4.83)	< 0.001	− 4.60 (− 4.91, − 4.29)	< 0.001	− 5.38 (− 5.71, − 5.05)	< 0.001	− 6.18 (− 6.57, − 5.80)	< 0.001
Per 10 μg/m^3^	− 2.68 (− 2.80, − 2.57)	< 0.001	− 2.31 (− 2.47, − 2.16)	< 0.001	−2.87 (− 3.04, − 2.70)	< 0.001	− 3.36 (− 3.57, − 3.16)	< 0.001
**MMEF**								
2nd quartile	− 3.16 (− 3.38, − 2.94)	< 0.001	− 2.13 (− 2.45, − 1.80)	< 0.001	− 2.88 (− 3.23, − 2.54)	< 0.001	− 3.92 (− 4.31, − 3.54)	< 0.001
3rd quartile	− 4.65 (− 4.90, − 4.40)	< 0.001	− 3.45 (− 3.82, − 3.08)	< 0.001	− 5.02 (− 5.42, − 4.62)	< 0.001	− 6.24 (− 6.69, − 5.78)	< 0.001
4th quartile	− 6.34 (− 6.65, − 6.02)	< 0.001	− 4.68 (− 5.14, − 4.22)	< 0.001	− 6.41 (− 6.90, − 5.91)	< 0.001	− 7.91 (− 8.46, − 7.35)	< 0.001
Per 10 μg/m^3^	− 3.30 (− 3.46, − 3.13)	< 0.001	− 2.56 (− 2.79, − 2.33)	< 0.001	− 3.03 (− 3.28, − 2.78)	< 0.001	− 3.81 (− 4.11, − 3.51)	< 0.001

Lung function was logarithmically transformed to normalise the data for analysis and then the original scale was transformed back to present the effects as percentage (%) difference in lung function parameters with 95% confidence interval for every 10 MET-h increase in PA or with inactive PA as the reference and for each 10 μg/m^3^ increase of PM_2.5_ or with the first quartile of the PM_2.5_ as the reference

All results were fully adjusted for age, sex, educational level, body mass index, season, year, physical labour at work, smoking, drinking, vegetable intake, fruit intake, occupational exposure to dust and organic solvent, hypertension, diabetes, dyslipidaemia, self-reported cardiovascular disease, and self-reported cancer

The 1^st^, 2^nd^, 3^rd^, and 4^th^ quartile of PM_2.5_ was < 21.67, 21.67–24.14, 24.14–28.81, and ≥ 28.81 μg/m^3^, respectively

*PA* physical activity, *FVC* forced vital capacity, *FEV*_*1*_ forced expiratory volume in 1 s, *MMEF* maximum mid-expiratory flow

**Fig. 2:**
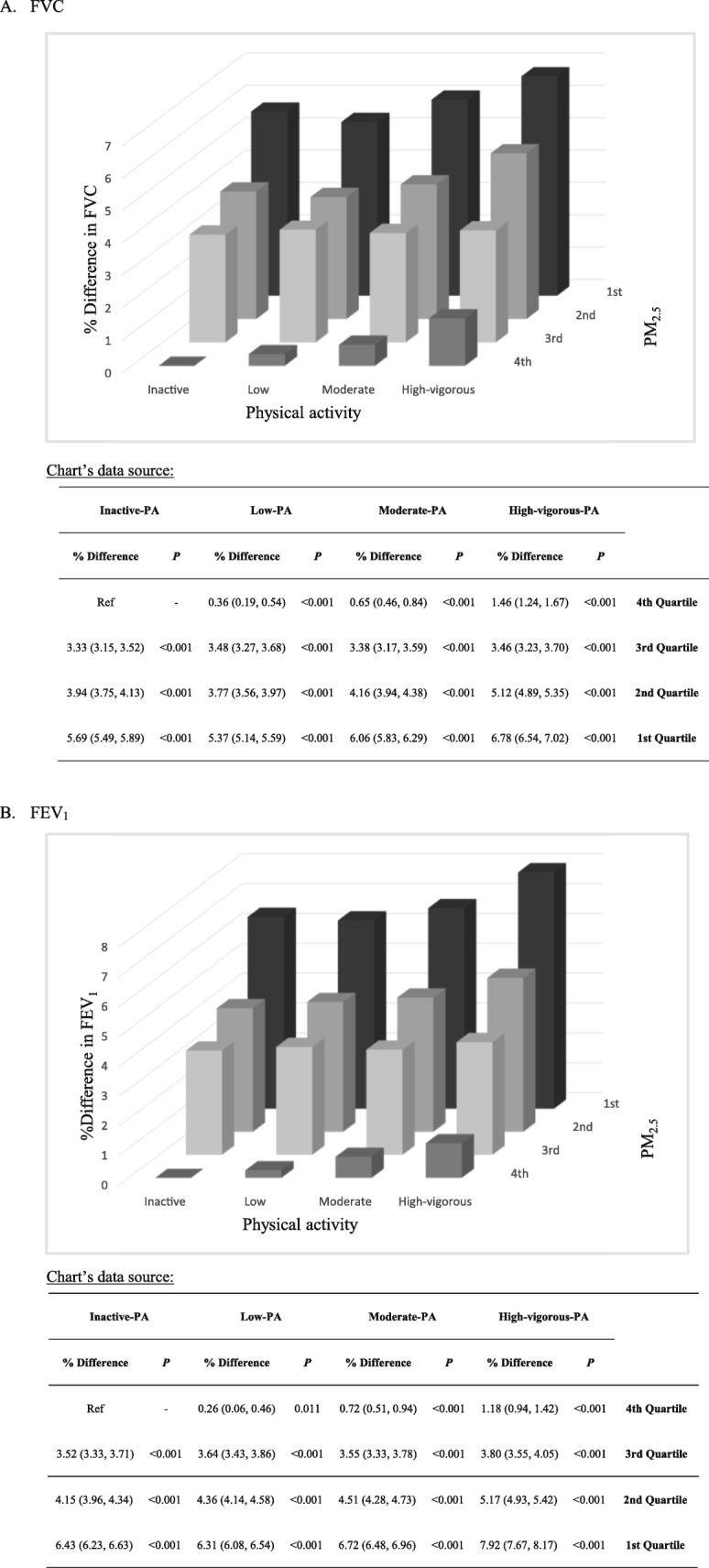

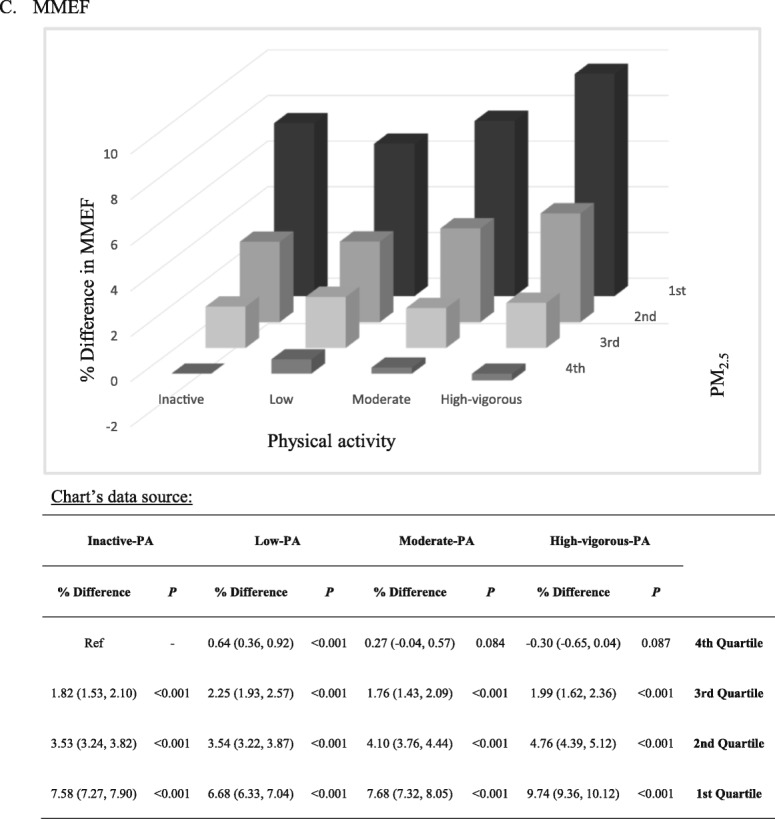
Continued.

The combined effects of PM_2.5_ exposure and PA are presented in Fig. 2. Participants with exposure to the 1st quartile of PM_2.5_ and the high PA had the best lung function. PM_2.5_ quartile was associated with a remarkable decreasing trend in lung function in each PA category. However, the increasing trends associated with PA were relatively flat.

To examine if habitual PA is harmful in the participants with a high PM_2.5_ exposure, we further compared the effects of different PA levels in participants in the lowest and highest decile of PM_2.5_ exposure (Table 4). The levels of PA benefits were generally larger in the low-exposure group, especially for participants with high PA and for MMEF. In the highest decile-exposure subgroup, benefits were observed for FVC and FEV_1_, but the statistical significance for MMEF disappeared when PA increased to the second quartile of high PA, and no statistical significance was observed for each 10 MET-h increase in PA (*P* = 0.125).

**Table 4:** Effects of habitual physical activity on lung function in Taiwanese adults in the lowest and highest 10% of PM_2.5_ exposure

Leisure time physical activity	N = 56,740	Low PM_**2.5**_ (≤ 10th %)^a^		N = 56,757	High PM_2.5_ (≥ 90th %)^a^	
		% difference	*P*		% difference	*P*
**FVC**						
Low PA	10,324	0.21 (− 0.10, 0.52)	0.192	12,022	0.26 (− 0.02, 0.54)	0.072
Moderate PA	10,946	0.50 (0.17, 0.83)	0.003	11,470	0.41 (0.09, 0.72)	0.010
High PA^b^						
1st quartile (16.5–22.8)	2777	1.27 (0.72, 1.81)	< 0.001	1940	0.76 (0.13, 1.40)	0.018
2nd quartile (22.8–24.8)	1343	0.84 (0.14, 1.56)	0.019	1433	1.06 (0.41, 1.71)	0.001
3rd quartile (24.8–35.8)	3062	1.12 (0.60, 1.64)	< 0.001	2802	0.58 (0.06, 1.10)	0.030
4th quartile (≥ 35.8)	3105	1.82 (1.29, 2.36)	< 0.001	2028	0.92 (0.33, 1.52)	0.002
Per 10 MET-h	–	0.27 (0.20, 0.35)	< 0.001	–	0.22 (0.14, 0.29)	< 0.001
**FEV**_**1**_						
Low PA	10,324	0.20 (− 0.11, 0.51)	0.209	12,022	0.05 (− 0.18, 0.29)	0.657
Moderate PA	10,946	0.60 (0.26, 0.93)	< 0.001	11,470	0.48 (0.22, 0.75)	< 0.001
High PA^b^						
1st quartile (16.5–22.8)	2777	1.38 (0.83, 1.94)	< 0.001	1940	1.35 (0.82, 1.88)	< 0.001
2nd quartile (22.8–24.8)	1343	0.72 (0.01, 1.44)	0.048	1433	0.95 (0.41, 1.49)	0.001
3rd quartile (24.8–35.8)	3062	1.54 (1.01, 2.07)	< 0.001	2802	1.11 (0.67, 1.55)	< 0.001
4th quartile (≥ 35.8)	3105	1.59 (1.05, 2.14)	< 0.001	2028	1.06 (0.56, 1.57)	< 0.001
10 MET-h	–	0.29 (0.21, 0.37)	< 0.001	–	0.21 (0.13, 0.29)	< 0.001
**MMEF**						
Low PA	10,324	− 0.06 (−0.54, 0.42)	0.801	12,022	0.17 (− 0.22, 0.56)	0.392
Moderate PA	10,946	0.49 (− 0.02, 1.00)	0.059	11,470	0.53 (0.09, 0.97)	0.019
High PA^b^						
1st quartile (16.5–22.8)	2777	2.06 (1.21, 2.91)	< 0.001	1940	1.69 (0.81, 2.57)	< 0.001
2nd quartile (22.8–24.8)	1343	1.50 (0.39, 2.62)	0.008	1433	0.60 (− 0.29, 1.49)	0.189
3rd quartile (24.8–35.8)	3062	1.91 (1.10, 2.72)	< 0.001	2802	0.44 (− 0.29, 1.17)	0.243
4th quartile (≥ 35.8)	3105	1.37 (0.54, 2.20)	0.001	2028	0.52 (− 0.31, 1.36)	0.221
10 MET-h	–	0.42 (0.30, 0.54)	< 0.001	–	0.10 (− 0.03, 0.23)	0.125

Lung function was logarithmically transformed to normalise the data for analysis, and then the original scale was transformed back to present the effects as percentage (%) difference in lung function parameters with 95% confidence interval

Participants with inactive PA category was the reference group

The results were fully adjusted for age, sex, educational level, body mass index, season, year, smoking, drinking, vegetable intake, fruit intake, occupational exposure to dust and organic solvent, hypertension, diabetes, hyperlipidaemia, self-reported cardiovascular disease, and self-reported cancer

*PA* physical activity, *FVC* forced vital capacity, *FEV*_*1*_ forced expiratory volume in 1 s, and *MMEF* maximum mid-expiratory flow

^a^The 10^th^ and 90^th^ PM_2.5_ refers to ≤ 19.6 and ≥ 39.9 μg/m^3^, respectively

^b^High PA was further categorised into four quartiles according to the MET-h to show the effects of very high PA levels

Sensitivity analyses generally yielded similar results (Tables S1-7 in Additional file: Table S1-Baseline combined associations. Table S2-The combined associations in participants with repeated measurements. Table S3-The combined associations in participants without lung-related diseases. Table S4-The combined associations in participants aged ≥ 25 years old. Table S5-The combined associations in participants who provided residential addresses. Table S6-The combined associations in ever-smokers. Table S7-The combined associations by adjusting different covariates).

## Discussion

To the best of our knowledge, this is the largest longitudinal cohort study that investigated the combined effects of long-term exposure to PM_2.5_ and habitual PA on lung function in adults. We found significant negative interaction effects between PA and PM_2.5_ in this Taiwanese population with an annual average PM_2.5_ exposure of 26.6 μg/m^3^. But habitual PA was generally associated with a better lung function in people exposed to different levels of PM_2.5_ in Taiwan.

This study has several important strengths. First, the nature of the cohort allowed us to longitudinally investigate the combined effects of PM_2.5_ exposure and PA on lung function. The large sample size and longitudinal design provided sufficient power to detect relatively small effects and resulted in more stable and precise estimates. Second, we collected detailed information on PA and a wide range of potential confounders/modifiers. The effect of physical labour at work was also considered. Finally, we used a spatiotemporal model to estimate PM_2.5_ exposure at a high resolution. This approach enables us to capture a high-resolution exposure and overcome the spatial coverage and interpolation problems that occur when using only data from monitoring stations.

The positive associations between PA and lung function have been well documented [28–30]. The negative associations between long-term exposure to PM_2.5_ and lung function were also reported by several large cohort studies [1, 17, 31, 32]. However, data on the combined health effects of long-term exposure to air pollution and habitual PA is relatively scarce. Most previous studies were conducted in Western countries, and the results were inconsistent. A recent study showed that the benefits of PA on mortality were not affected by air pollution [3]. The hazard ratios (HRs) of respiratory mortality ranged from 0.74 to 1.50, as compared with the participants exposed to a low level of PM_2.5_ (< 35.3 μg/m^3^) and had a high PA (≥ 21.0 MET-h/week). In a Danish cohort, Andersen et al. [12] and Fisher et al. [13] used mortality and asthma/COPD hospitalisation as health outcomes and found that the effects of PA and air pollution were generally independent. Andersen et al. [12] further found that only the benefits derived from cycling and gardening on respiratory mortality were slightly larger for participants exposed to moderate/low NO_2_ than that for those exposed to high NO_2_. They concluded that the benefits of PA outweighed the harmfulness of air pollution on mortality [12], whilst air pollution did not reduce the benefits of PA for asthma/COPD hospitalisation [13]. The ECRHS study [30] reported that PA had beneficial effects on lung function in current smokers, irrespective of air pollution levels. Our study also had similar findings (i.e. PA benefits remained in people with different exposure to PM_2.5_) although we targeted a general population. Nevertheless, the ECRHS study did not find significant interaction effects, which was different from our study. The study by Cole-Hunter et al. [2] showed that daily physical activity was not a mediator for a range of cardiopulmonary outcomes including blood pressure, pulse, heart rate variability, and lung function. In contrast, we found that PA was a modifier on the association between air pollution and lung function. Our findings are in line with the study by McConnell et al. [14] and the PASTA study [15]*.* McConnell et al. [14] reported that playing sports was associated with a higher risk of incident asthma [relative risk (RR) (95%CI) was 3.3 (95%CI 1.9–5.8)] in areas of high ozone pollution, but not in those with low ozone concentration. The PASTA study [15] observed a negative interaction effect between black carbon and PA on lung function, and the estimated reductions in lung function ranged from 0.08 to 11.70 ml. Other studies focused on some special populations. For example, Garcia-Aymerich et al. [16] reported PA might reduce lung function decrease among active smokers by 2.6–7.7 ml/year. The study by Endes et al. [33] showed PA might protect against the adverse effects of air pollution on arterial stiffness in elderly, and the odds ratio for the interaction between PA and PM_2.5_ was 0.79 (95%CI 0.6–1.04). It is difficult to directly compare our study with previous studies because the study design, targeted population, health outcome, PA definition, and air pollution level/source are quite different.

Several other studies reported that health benefits of habitual PA outweighed harmfulness of air pollution, and they generally used modelling methods based on literature-derived risk of air pollution and literature-derived benefits of PA [34–38]. These studies assumed that the effects of PA and air pollution were independent and generally focused on active travel and used mortality or life expectancy as health outcomes. Our study provided direct evidence on the combined effects of PA and PM_2.5_ on lung function.

The research findings also differ somewhat from those of our previous study that investigated the combined effects of PA and PM_2.5_ on systemic inflammation based on the same cohort [22]. In that study, we found that the effects of PA and PM_2.5_ were generally independent and that the benefits of PA slightly outweighed the harmfulness of PM_2.5_. We speculate that the differences are possibly due to the different biological mechanisms in PM_2.5_ effects on lung function and systemic inflammation. Pollutants may directly affect the pulmonary system, whilst for cardiovascular systems, the effect is indirect and might be associated with much more complicated processes. Nonetheless, more research is warranted for the combined effects on different health outcomes.

Our results show that the increased intake of PM_2.5_ due to PA may attenuate the benefits of habitual PA on lung function, but significant beneficial effects were still observed across the PM_2.5_ quartiles (Tables 2 and 3), even among the participants in the top decile of exposure level (except for MMEF, Table 4). In consideration of the benefits of PA on lung function and other positive health effects of PA, our results suggest that in general, it is not necessary to reduce habitual PA even for people who live in relatively highly polluted areas. Our results also suggest that we can maximise the PA beneficial effects by mitigating air pollution. Thus, it is valuable to improve urban and transport planning [39, 40] and promote safe active mobility (such as bicycling in light polluted days), which may increase PA and reduce pollutant emissions [41]*.* E-mobility and indoor PA should also be promoted to improve pulmonary health [42].

Table 4 shows that people with very high levels of both PA and PM_2.5_ had insignificant PA benefits on MMEF, implying that people living in areas with high levels of air pollution should be cautious when undertaking an extremely high volume of outdoor PA. However, the PA benefits remained on FVC and FEV_1_. It was difficult to identify the precise levels of PA and PM_2.5_ about which people should be cautious, because the number of participants with very high levels of both PA and PM_2.5_ was small in this study. Further studies on this topic in participants with very high levels of both PA and PM_2.5_ are needed.

Although measurements of PA and PM_2.5_ were different, the association between lung function and PM_2.5_ exposure seemed much stronger than the association between lung function, and habitual PA [each IQR (7.14 μg/m^3^) increment in PM_2.5_ was associated with a reduction of 1.65%, 1.99%, and 2.28% in FVC, FEV_1_, and MMEF, respectively, whilst each IQR (10.94 MET-h) increment in PA was associated with an increase of 0.25%, 0.20%, and 0.27% in FVC, FEV_1_, and MMEF, respectively]; similar patterns were observed when PA and PM_2.5_ were treated as categorical variables (Fig. 2). This finding suggests the importance of air pollution mitigation in the protection of pulmonary health.

The mechanism of the interaction effects on lung function remains unclear. Previous studies show that regular PA enhances the production of anti-inflammatory markers and simultaneously constrains the production of inflammatory markers [43, 44]. Conversely, PM_2.5_ is associated with a higher level of inflammation and oxidative stress [45]. Clearly, higher levels of PA may increase the inhalation of particles, which will elevate pulmonary inflammation. It follows that the higher levels of PA present in highly polluted areas may suppress the benefits of PA on lung function. In addition, body weight or BMI may also play a role in the pathway of interaction because both PA and lung function are related to body weight or BMI, and a previous study shows that people who were exposed to low PM_2.5_ concentrations and undertook moderate PA generally had a lower body weight [46]. Nonetheless, it is not clear how body weight or BMI mediates the pathway. Further studies on the mechanism are warranted.

This study has several limitations. First, we did not distinguish between PA that took place indoors and outdoors. Thus, we could not evaluate outdoor PA exclusively. However, only a small portion (7.3%) of residents reported indoor activities as their most frequent PA in the 2015 national activity survey [47]. Second, we excluded 64,561 participants in this study and 25,908 (7.6%) of them were due to FEV_1_/FVC ≥ 100%. It is difficult to conduct lung function test in large-scale studies, and many factors might contribute to the unsuccessful test such as participant cooperation, technician skill, and instrument quality. However, we collected the data from a standard and routine medical screening programme [17, 48], and there are no evidence showing that participants with an unsuccessful spirometry are more likely associated with the levels of habitual PA and PM_2.5_ exposure. Therefore, the exclusion is unlikely to have resulted in any bias. Third, we did not consider indoor air pollution. However, we accounted for smoking, which is one of the most important sources of household air pollution in developed economies. Fourth, the PM_2.5_ exposure levels were assigned to the participants’ fixed addresses and did not consider daily activity patterns. More advanced technologies that can provide more accurate assessments of exposure are needed in future studies. In addition, information on other gaseous pollutants such as ozone, NO_2_, and SO_2_ was not available, and thus, we could not isolate the potential effects of these pollutants. The generally high correlations between gaseous pollutants and PM_2.5_ do suggest that they should be analysed separately. Finally, the participants in this study were generally well-educated (61.8% of the participants had an education of college or above in this study, whilst it was approximately 36.8% among people with an age of ≥ 15 years old in Taiwan in 2010 [49]) and had a relatively higher level of PA (30.4% of the participants had a medium or high PA in this study, whilst the National Health Interview Survey showed 11–14% of the people met the national recommendations [50]). Therefore, we should be cautious to generalise the findings to general population.

## Conclusions

In summary, we observed a significant negative interaction effect between long-term exposure to PM_2.5_ and habitual PA in this Taiwan adult population with a relatively high exposure to PM_2.5_, showing the increased intake of PM_2.5_ due to PA may attenuate the benefits of habitual PA on lung function. But habitual PA was generally associated with a better lung function in people with different exposure to PM_2.5_. Habitual PA may still be recommended to people residing in relatively polluted regions. However, further research is warranted to validate our findings in regions with higher levels of air pollution than Taiwan. Our results reinforce the importance of air pollution mitigation, i.e. reduce the harmful effects of air pollution and maximise the beneficial effects of habitual PA.

## Supplementary information

**Additional file 1: Figure S1.** The flow chart of participants selection. **Table S1**. Comparison on lung function at baseline with different levels of physical activity and PM_2.5_ exposures (*N*=278,065). Abbreviations: PA, physical activity; FVC, forced vital capacity; FEV_1_, forced expiratory volume in 1 second; MMEF, maximum mid-expiratory flow. Lung function was logarithmically transformed to normalise the data for analysis and then the original scale was transformed back to present the effects as percentage (%) difference in lung function parameters with 95% confidence interval. Results were fully adjusted for age, sex, educational level, body mass index, season, year, physical labour at work, smoking, drinking, vegetable intake, fruit intake, occupational exposure to dust & organic solvent, hypertension, diabetes, dyslipidemia, self-reported cardiovascular disease and self-reported cancer. The 1st, 2nd, 3rd and 4th quartile of PM_2.5_ was <21.59, 21.59-24.00, 24.00-29.39 and ≥29.39 μg/m^3^ respectively. **Table S2**. Comparison on lung function with different levels of physical activity and PM_2.5_ exposures in the participants with more than one medical examinations (*N*=405,451). Abbreviations: PA, physical activity; FVC, forced vital capacity; FEV_1_, forced expiratory volume in 1 second; MMEF, maximum mid-expiratory flow. Lung function was logarithmically transformed to normalise the data for analysis and then the original scale was transformed back to present the effects as percentage (%) difference in lung function parameters with 95% confidence interval. Results were fully adjusted for age, sex, educational level, body mass index, season, year, physical labour at work, smoking, drinking, vegetable intake, fruit intake, occupational exposure to dust & organic solvent, hypertension, diabetes, dyslipidemia, self-reported cardiovascular disease and self-reported cancer. The 1st, 2nd, 3rd and 4th quartile of PM_2.5_ was <21.70, 21.70-24.16, 24.16-28.57 and ≥28.57 μg/m^3^ respectively. **Table S3**. Comparison on lung function with different levels of physical activity and PM_2.5_ exposures for the healthy participants without prior lung-related diseases (asthma, chronic obstructive pulmonary diseases, and cancer) (*N*=538,678). Abbreviations: PA, physical activity; FVC, forced vital capacity; FEV_1_, forced expiratory volume in 1 second; MMEF, maximum mid-expiratory flow. Lung function was logarithmically transformed to normalise the data for analysis and then the original scale was transformed back to present the effects as percentage (%) difference in lung function parameters with 95% confidence interval. Results were fully adjusted for age, sex, educational level, body mass index, season, year, physical labour at work, smoking, drinking, vegetable intake, fruit intake, occupational exposure to dust & organic solvent, hypertension, diabetes, dyslipidemia, self-reported cardiovascular disease and self-reported cancer. The 1st, 2nd, 3rd and 4th quartile of PM_2.5_ was <21.68, 21.68-24.16, 24.16-28.96 and ≥28.96 μg/m^3^ respectively. **Table S4**. Comparison on lung function of healthy Taiwanese adults older than 25 years old with different levels of physical activity and PM_2.5_ exposures (*N*=548,811). Abbreviations: PA, physical activity; FVC, forced vital capacity; FEV_1_, forced expiratory volume in 1 second; MMEF, maximum mid-expiratory flow. Lung function was logarithmically transformed to normalise the data for analysis and then the original scale was transformed back to present the effects as percentage (%) difference in lung function parameters with 95% confidence interval. Results were fully adjusted for age, sex, educational level, body mass index, season, year, physical labour at work, smoking, drinking, vegetable intake, fruit intake, occupational exposure to dust & organic solvent, hypertension, diabetes, dyslipidemia, self-reported cardiovascular disease and self-reported cancer. The 1st, 2nd, 3rd and 4th quartile of PM_2.5_ was <21.68, 21.68-24.14, 24.14-28.76 and ≥28.76 μg/m^3^ respectively. **Table S5**. Comparison on lung function with different levels of physical activity and PM_2.5_ exposures for the participants providing only residential addresses (*N*=567,557). Abbreviations: PA, physical activity; FVC, forced vital capacity; FEV_1_, forced expiratory volume in 1 second; MMEF, maximum mid-expiratory flow. Lung function was logarithmically transformed to normalise the data for analysis and then the original scale was transformed back to present the effects as percentage (%) difference in lung function parameters with 95% confidence interval. Results were fully adjusted for age, sex, educational level, body mass index, season, year, physical labour at work, smoking, drinking, vegetable intake, fruit intake, occupational exposure to dust & organic solvent, hypertension, diabetes, dyslipidemia, self-reported cardiovascular disease and self-reported cancer. The 1st, 2nd, 3rd and 4th quartile of PM_2.5_ was <21.56, 21.56-24.03, 24.03-29.76 and ≥29.76 μg/m^3^ respectively. **Table S6**. Comparison on lung function of non-smokers with different levels of physical activity and PM_2.5_ exposures (*N*=423,491). Abbreviations: PA, physical activity; FVC, forced vital capacity; FEV_1_, forced expiratory volume in 1 second; MMEF, maximum mid-expiratory flow. Lung function was logarithmically transformed to normalise the data for analysis and then the original scale was transformed back to present the effects as percentage (%) difference in lung function parameters with 95% confidence interval. Results were fully adjusted for age, sex, educational level, body mass index, season, year, physical labour at work, smoking, drinking, vegetable intake, fruit intake, occupational exposure to dust & organic solvent, hypertension, diabetes, dyslipidemia, self-reported cardiovascular disease and self-reported cancer. The 1st, 2nd, 3rd and 4th quartile of PM_2.5_ was <21.70, 21.70-24.17, 24.17-29.22 and ≥29.22 μg/m^3^ respectively. **Table S7**. Associations of lung function with habitual physical activity and PM_2.5_ exposure in Taiwanese adults (*N*=567,557). Abbreviations: PA, physical activity; FVC, forced vital capacity; FEV_1_, forced expiratory volume in 1 second; MMEF, maximum mid-expiratory flow. Lung function was logarithmically transformed to normalise the data for analysis and then the original scale was transformed back to present the effects as percentage (%) difference in lung function parameters with 95% confidence interval. The effects were presented as % difference in lung function with 95% confidence level. Participants who were in inactive-PA category or in the 1st quartile of PM_2.5_ comprised the reference group. The 1st, 2nd, 3rd and 4th quartile of PM_2.5_ was <21.67, 21.67-24.14, 24.14-28.81 and ≥28.81 μg/m^3^, respectively. ^a^ Model 1: adjusted for age, sex, educational level, body mass index, season, year, physical labour at work, smoking, drinking, vegetable intake and fruit intake; Model 2 further adjusted for occupational exposure to dust & organic solvent.

## Data Availability

The anonymised data are available upon appropriate request from the corresponding author.
