# Supplementary material for: Does fine particulate matter (PM2.5) affect the benefits of habitual physical activity on lung function in adults: a longitudinal cohort study
Source: BMC Med. 2020 May 13;18:134. doi: 10.1186/s12916-020-01570-5 (PMC7218575; doi:10.1186/s12916-020-01570-5)
Supplement: Supplementary file 1 — Additional file 1: Figure S1. The flow chart of participants selection. Table S1. Comparison on lung function at baseline with different levels of physical activity and PM2.5 exposures (N=278,065). Abbreviations: PA, physical activity; FVC, forced vital capacity; FEV1, forced expiratory volume in 1 second; MMEF, maximum mid-expiratory flow. Lung function was logarithmically transformed to normalise the data for analysis and then the original scale was transformed back to present the effects as percentage (%) difference in lung function parameters with 95% confidence interval. Results were fully adjusted for age, sex, educational level, body mass index, season, year, physical labour at work, smoking, drinking, vegetable intake, fruit intake, occupational exposure to dust & organic solvent, hypertension, diabetes, dyslipidemia, self-reported cardiovascular disease and self-reported cancer. The 1st, 2nd, 3rd and 4th quartile of PM2.5 was <21.59, 21.59-24.00, 24.00-29.39 and ≥29.39 μg/m3 respectively. Table S2. Comparison on lung function with different levels of physical activity and PM2.5 exposures in the participants with more than one medical examinations (N=405,451). Abbreviations: PA, physical activity; FVC, forced vital capacity; FEV1, forced expiratory volume in 1 second; MMEF, maximum mid-expiratory flow. Lung function was logarithmically transformed to normalise the data for analysis and then the original scale was transformed back to present the effects as percentage (%) difference in lung function parameters with 95% confidence interval. Results were fully adjusted for age, sex, educational level, body mass index, season, year, physical labour at work, smoking, drinking, vegetable intake, fruit intake, occupational exposure to dust & organic solvent, hypertension, diabetes, dyslipidemia, self-reported cardiovascular disease and self-reported cancer. The 1st, 2nd, 3rd and 4th quartile of PM2.5 was <21.70, 21.70-24.16, 24.16-28.57 and ≥28.57 μg/m3 respectively [file 12916_2020_1570_MOESM1_ESM.doc]

**Does Fine Particulate Matter (PM_2.5_) Affect the Benefits of Habitual Physical Activity on Lung Function in Adults:**

**A Longitudinal Cohort Study**

Cui Guo; Yacong Bo; Ta-Chien Chan; Zilong Zhang; Changqing Lin; Tony Tam; Alexis K.H. Lau; Ly-yun Chang; Gerard Hoek; Xiang Qian Lao

**Additional file**

**Figure S1. The flow chart of participants selection.**

342,626 participants with spirometric tests

248,350 participants were excluded:

158,543 joined before 2001

10,435 were younger than 20 years old

79,372 had no spirometric tests

278,065 participants with 567,557 observations

A total of 590,976 participants from the original dataset in 1996-2014

64,561 had incomplete information:

2,668 on PM_2.5_ concentration

35,985 on other covariates

25,908 with FEV_1_:FVC ≥ 100

**Table S1. Comparison on lung function at baseline with different levels of physical activity and PM_2.5_ exposures (N=278,065).**

Abbreviations: PA, physical activity; FVC, forced vital capacity; FEV_1_, forced expiratory volume in 1 second; MMEF, maximum mid-expiratory flow.

Lung function was logarithmically transformed to normalise the data for analysis and then the original scale was transformed back to present the effects as percentage (%) difference in lung function parameters with 95% confidentce interval.

Results were fully adjusted for age, sex, educational level, body mass index, season, year, physical labour at work, smoking, drinking, vegetable intake, fruit intake, occupational exposure to dust & organic solvent, hypertension, diabetes, hyperlipidemia, self-reported cardiovascular disease and self-reported cancer.

The 1^st^, 2^nd^, 3^rd^ and 4^th^ quartile of PM_2.5_ was <21.59, 21.59-24.00, 24.00-29.39 and ≥29.39 μg/m³ respectively.

| **Lung function** | **Inactive-PA** | | **Low-PA** | | **Moderate-PA** | | **High-PA** | |
| --- | --- | --- | --- | --- | --- | --- | --- | --- |
|  | **% Difference** | ***P*** | **% Difference** | ***P*** | **% Difference** | ***P*** | **% Difference** | ***P*** |
| **FVC** |  |  |  |  |  |  |  |  |
| 4th Quartile | Ref | - | 0.52 (0.17, 0.87) | 0.003 | 1.16 (0.78, 1.53) | <0.001 | 1.46 (1.05, 1.87) | <0.001 |
| 3rd Quartile | 4.09 (3.82, 4.36) | <0.001 | 4.52 (4.18, 4.87) | <0.001 | 5.24 (4.86, 5.62) | <0.001 | 6.28 (5.85, 6.71) | <0.001 |
| 2nd Quartile | 4.35 (4.07, 4.62) | <0.001 | 4.43 (4.09, 4.78) | <0.001 | 5.06 (4.68, 5.44) | <0.001 | 6.60 (6.19, 7.02) | <0.001 |
| 1st Quartile | 4.97 (4.70, 5.25) | <0.001 | 5.63 (5.27, 5.99) | <0.001 | 6.15 (5.76, 6.54) | <0.001 | 7.34 (6.94, 7.74) | <0.001 |
| **FEV_1_** |  |  |  |  |  |  |  |  |
| 4th Quartile | Ref | - | 0.63 (0.28, 0.98) | <0.001 | 1.02 (0.64, 1.40) | <0.001 | 0.96 (0.55, 1.37) | <0.001 |
| 3rd Quartile | 4.59 (4.32, 4.87) | <0.001 | 4.86 (4.52, 5.21) | <0.001 | 5.45 (5.06, 5.83) | <0.001 | 6.07 (5.64, 6.51) | <0.001 |
| 2nd Quartile | 4.60 (4.33, 4.88) | <0.001 | 4.76 (4.41, 5.11) | <0.001 | 5.08 (4.70, 5.47) | <0.001 | 6.21 (5.80, 6.63) | <0.001 |
| 1st Quartile | 5.55 (5.27, 5.82) | <0.001 | 5.96 (5.60, 6.33) | <0.001 | 6.50 (6.11, 6.90) | <0.001 | 7.38 (6.98, 7.79) | <0.001 |
| **MMEF** |  |  |  |  |  |  |  |  |
| 4th Quartile | Ref | - | 1.07 (0.53, 1.61) | <0.001 | 0.70 (0.12, 1.28) | 0.017 | -0.19 (-0.81, 0.44) | 0.554 |
| 3rd Quartile | 3.77 (3.36, 4.19) | <0.001 | 3.51 (2.98, 4.03) | <0.001 | 3.44 (2.86, 4.02) | <0.001 | 3.27 (2.61, 3.92) | <0.001 |
| 2nd Quartile | 3.64 (3.22, 4.06) | <0.001 | 3.65 (3.12, 4.18) | <0.001 | 3.47 (2.89, 4.06) | <0.001 | 3.96 (3.33, 4.60) | <0.001 |
| 1st Quartile | 5.72 (5.29, 6.14) | <0.001 | 5.73 (5.18, 6.29) | <0.001 | 5.66 (5.07, 6.26) | <0.001 | 6.21 (5.59, 6.83) | <0.001 |

**Table S2. Comparison on lung function with different levels of physical activity and PM_2.5_ exposures in the participants with more than one medical examinations (N=405,451).**

Abbreviations: PA, physical activity; FVC, forced vital capacity; FEV_1_, forced expiratory volume in 1 second; MMEF, maximum mid-expiratory flow.

Lung function was logarithmically transformed to normalise the data for analysis and then the original scale was transformed back to present the effects as percentage (%) difference in lung function parameters with 95% confidentce interval.

Results were fully adjusted for age, sex, educational level, body mass index, season, year, physical labour at work, smoking, drinking, vegetable intake, fruit intake, occupational exposure to dust & organic solvent, hypertension, diabetes, hyperlipidemia, self-reported cardiovascular disease and self-reported cancer.

The 1^st^, 2^nd^, 3^rd^ and 4^th^ quartile of PM_2.5_ was <21.70, 21.70-24.16, 24.16-28.57 and ≥28.57 μg/m³ respectively.

| **Lung function** | **Inactive-PA** | | **Low-PA** | | **Moderate-PA** | | **High-PA** | |
| --- | --- | --- | --- | --- | --- | --- | --- | --- |
|  | **% Difference** | ***P*** | **% Difference** | ***P*** | **% Difference** | ***P*** | **% Difference** | ***P*** |
| **FVC** |  |  |  |  |  |  |  |  |
| 4th Quartile | Ref | - | 0.42 (0.22, 0.63) | <0.001 | 0.73 (0.51, 0.95) | <0.001 | 1.31 (1.06, 1.56) | <0.001 |
| 3rd Quartile | 2.88 (2.66, 3.10) | <0.001 | 3.23 (2.99, 3.48) | <0.001 | 3.24 (2.99, 3.50) | <0.001 | 3.38 (3.11, 3.66) | <0.001 |
| 2nd Quartile | 3.97 (3.74, 4.20) | <0.001 | 3.87 (3.62, 4.12) | <0.001 | 4.19 (3.93, 4.45) | <0.001 | 4.95 (4.67, 5.22) | <0.001 |
| 1st Quartile | 5.78 (5.54, 6.02) | <0.001 | 5.32 (5.06, 5.59) | <0.001 | 5.88 (5.61, 6.15) | <0.001 | 6.62 (6.34, 6.91) | <0.001 |
| **FEV_1_** |  |  |  |  |  |  |  |  |
| 4th Quartile | Ref | - | 0.42 (0.22, 0.62) | <0.001 | 0.69 (0.47, 0.90) | <0.001 | 0.98 (0.74, 1.23) | <0.001 |
| 3rd Quartile | 3.38 (3.15, 3.60) | <0.001 | 3.50 (3.26, 3.75) | <0.001 | 3.21 (2.96, 3.47) | <0.001 | 3.59 (3.32, 3.87) | <0.001 |
| 2nd Quartile | 4.32 (4.09, 4.55) | <0.001 | 4.27 (4.02, 4.52) | <0.001 | 4.58 (4.32, 4.84) | <0.001 | 5.52 (5.24, 5.79) | <0.001 |
| 1st Quartile | 6.71 (6.46, 6.96) | <0.001 | 6.32 (6.05, 6.59) | <0.001 | 6.72 (6.45, 7.00 | <0.001 | 8.10 (7.81, 8.39) | <0.001 |
| **MMEF** |  |  |  |  |  |  |  |  |
| 4th Quartile | Ref | - | 0.59 (0.26, 0.92) | <0.001 | 0.19 (-0.17, 0.54) | 0.298 | -0.45 (-0.85, -0.05) | 0.029 |
| 3rd Quartile | 2.01 (1.66, 2.36) | <0.001 | 2.26 (1.88, 2.65) | <0.001 | 1.88 (1.49, 2.28) | <0.001 | 2.14 (1.70, 2.57) | <0.001 |
| 2nd Quartile | 4.07 (3.71, 4.43) | <0.001 | 4.14 (3.74, 4.53) | <0.001 | 4.34 (3.93, 4.76) | <0.001 | 5.45 (5.01, 5.89) | <0.001 |
| 1st Quartile | 8.24 (7.85, 8.63) | <0.001 | 7.32 (6.89, 7.75) | <0.001 | 8.29 (7.85, 8.73) | <0.001 | 10.38 (9.92, 10.84) | <0.001 |

**Table S3. Comparison on lung function with different levels of physical activity and PM_2.5_ exposures for the healthy participants without prior lung-related diseases (asthma, chronic obstructive pulmonary diseases, and cancer) (N=538,678).**

Abbreviations: PA, physical activity; FVC, forced vital capacity; FEV_1_, forced expiratory volume in 1 second; MMEF, maximum mid-expiratory flow.

Lung function was logarithmically transformed to normalise the data for analysis and then the original scale was transformed back to present the effects as percentage (%) difference in lung function parameters with 95% confidentce interval.

Results were fully adjusted for age, sex, educational level, body mass index, season, year, physical labour at work, smoking, drinking, vegetable intake, fruit intake, occupational exposure to dust & organic solvent, hypertension, diabetes, hyperlipidemia, self-reported cardiovascular disease and self-reported cancer.

The 1^st^, 2^nd^, 3^rd^ and 4^th^ quartile of PM_2.5_ was <21.68, 21.68-24.16, 24.16-28.96 and ≥28.96 μg/m³ respectively.

| **Lung function** | **Inactive-PA** | | **Low-PA** | | **Moderate-PA** | | **High-PA** | |
| --- | --- | --- | --- | --- | --- | --- | --- | --- |
|  | **% Difference** | ***P*** | **% Difference** | ***P*** | **% Difference** | ***P*** | **% Difference** | ***P*** |
| **FVC** |  |  |  |  |  |  |  |  |
| 4th Quartile | Ref | - | 0.21 (0.04, 0.39) | 0.019 | 0.57 (0.38, 0.76) | <0.001 | 1.03 (0.81, 1.25) | <0.001 |
| 3rd Quartile | 3.08 (2.89, 3.26) | <0.001 | 3.32 (3.11, 3.53) | <0.001 | 3.22 (3.00, 3.43) | <0.001 | 3.87 (3.63, 4.11) | <0.001 |
| 2nd Quartile | 3.86 (3.67, 4.05) | <0.001 | 3.78 (3.57, 3.99) | <0.001 | 4.07 (3.86, 4.29) | <0.001 | 5.34 (5.10, 5.57) | <0.001 |
| 1st Quartile | 5.52 (5.32, 5.72) | <0.001 | 5.23 (5.00, 5.45) | <0.001 | 5.71 (5.48, 5.94) | <0.001 | 6.59 (6.35, 6.83) | <0.001 |
| **FEV_1_** |  |  |  |  |  |  |  |  |
| 4th Quartile | Ref | - | 0.44 (0.27, 0.62) | <0.001 | 0.72 (0.53, 0.91) | <0.001 | 1.07 (0.86, 1.29) | <0.001 |
| 3rd Quartile | 3.39 (3.20, 3.57) | <0.001 | 3.66 (3.45, 3.87) | <0.001 | 3.72 (3.51, 3.94) | <0.001 | 4.02 (3.78, 4.25) | <0.001 |
| 2nd Quartile | 4.42 (4.23, 4.61) | <0.001 | 4.34 (4.13, 4.55) | <0.001 | 4.68 (4.46, 4.90) | <0.001 | 5.60 (5.36, 5.83) | <0.001 |
| 1st Quartile | 6.64 (6.43, 6.84) | <0.001 | 6.48 (6.25, 6.70) | <0.001 | 6.99 (6.76, 7.22) | <0.001 | 8.17 (7.93, 8.41) | <0.001 |
| **MMEF** |  |  |  |  |  |  |  |  |
| 4th Quartile | Ref | - | 0.54 (0.26, 0.83) | <0.001 | 0.43 (0.13, 0.74) | 0.005 | -0.46 (-0.81, -0.11) | 0.009 |
| 3rd Quartile | 2.55 (2.27, 2.84) | <0.001 | 2.31 (1.99, 2.63) | <0.001 | 2.36 (2.03, 2.69) | <0.001 | 2.33 (1.96, 2.70) | <0.001 |
| 2nd Quartile | 3.92 (3.63, 4.21) | <0.001 | 4.19 (3.86, 4.52) | <0.001 | 4.10 (3.76, 4.44) | <0.001 | 5.08 (4.72, 5.45) | <0.001 |
| 1st Quartile | 7.95 (7.64, 8.26) | <0.001 | 7.16 (6.81, 7.52) | <0.001 | 8.01 (7.65, 8.37) | <0.001 | 9.97 (9.58, 10.35) | <0.001 |

**Table S4. Comparison on lung function of healthy Taiwanese adults older than 25 years old with different levels of physical activity and PM_2.5_ exposures (N=548,811).**

Abbreviations: PA, physical activity; FVC, forced vital capacity; FEV_1_, forced expiratory volume in 1 second; MMEF, maximum mid-expiratory flow.

Lung function was logarithmically transformed to normalise the data for analysis and then the original scale was transformed back to present the effects as percentage (%) difference in lung function parameters with 95% confidentce interval.

Results were fully adjusted for age, sex, educational level, body mass index, season, year, physical labour at work, smoking, drinking, vegetable intake, fruit intake, occupational exposure to dust & organic solvent, hypertension, diabetes, hyperlipidemia, self-reported cardiovascular disease and self-reported cancer.

The 1^st^, 2^nd^, 3^rd^ and 4^th^ quartile of PM_2.5_ was <21.68, 21.68-24.14, 24.14-28.76 and ≥28.76 μg/m³ respectively.

| **Lung function** | **Inactive-PA** | | **Low-PA** | | **Moderate-PA** | | **High-PA** | |
| --- | --- | --- | --- | --- | --- | --- | --- | --- |
|  | **% Difference** | ***P*** | **% Difference** | ***P*** | **% Difference** | ***P*** | **% Difference** | ***P*** |
| **FVC** |  |  |  |  |  |  |  |  |
| 4th Quartile | Ref | - | 0.37 (0.19, 0.55) | <0.001 | 0.72 (0.52, 0.91) | <0.001 | 1.11 (0.89, 1.33) | <0.001 |
| 3rd Quartile | 3.37 (3.18, 3.56) | <0.001 | 3.68 (3.47, 3.89) | <0.001 | 3.73 (3.52, 3.95) | <0.001 | 3.62 (3.38, 3.86) | <0.001 |
| 2nd Quartile | 4.08 (3.89, 4.27) | <0.001 | 3.96 (3.75, 4.18) | <0.001 | 4.48 (4.26, 4.70) | <0.001 | 5.41 (5.17, 5.64) | <0.001 |
| 1st Quartile | 5.73 (5.53, 5.93) | <0.001 | 5.37 (5.15, 5.60) | <0.001 | 6.09 (5.86, 6.32) | <0.001 | 7.05 (6.81, 7.29) | <0.001 |
| **FEV_1_** |  |  |  |  |  |  |  |  |
| 4th Quartile | Ref | - | 0.21 (0.01, 0.42) | 0.041 | 0.51 (0.29, 0.72) | <0.001 | 0.78 (0.54, 1.03) | <0.001 |
| 3rd Quartile | 3.70 (3.51, 3.90) | <0.001 | 3.75 (3.53, 3.97) | <0.001 | 3.86 (3.63, 4.09) | <0.001 | 3.87 (3.61, 4.12) | <0.001 |
| 2nd Quartile | 4.41 (4.22, 4.60) | <0.001 | 4.62 (4.40, 4.85) | <0.001 | 4.37 (4.14, 4.60) | <0.001 | 5.67 (5.42, 5.92) | <0.001 |
| 1st Quartile | 6.52 (6.32, 6.72) | <0.001 | 6.27 (6.03, 6.51) | <0.001 | 6.86 (6.62, 7.10) | <0.001 | 7.84 (7.58, 8.09) | <0.001 |
| **MMEF** |  |  |  |  |  |  |  |  |
| 4th Quartile | Ref | - | 0.70 (0.41, 1.00) | <0.001 | 0.02 (-0.29, 0.33) | 0.883 | -0.46 (-0.81, -0.10) | 0.011 |
| 3rd Quartile | 1.94 (1.65, 2.24) | <0.001 | 1.90 (1.57, 2.22) | <0.001 | 2.41 (2.07, 2.75) | <0.001 | 2.10 (1.72, 2.47) | <0.001 |
| 2nd Quartile | 3.78 (3.48, 4.08) | <0.001 | 3.74 (3.41, 4.08) | <0.001 | 4.02 (3.67, 4.37) | <0.001 | 5.21 (4.84, 5.58) | <0.001 |
| 1st Quartile | 7.42 (7.10, 7.74) | <0.001 | 6.65 (6.29, 7.01) | <0.001 | 7.88 (7.51, 8.25) | <0.001 | 9.49 (9.10, 9.88) | <0.001 |

**Table S5. Comparison on lung function with different levels of physical activity and PM_2.5_ exposures for the participants providing only residential addresses (N=567,557).**

Abbreviations: PA, physical activity; FVC, forced vital capacity; FEV_1_, forced expiratory volume in 1 second; MMEF, maximum mid-expiratory flow.

Lung function was logarithmically transformed to normalise the data for analysis and then the original scale was transformed back to present the effects as percentage (%) difference in lung function parameters with 95% confidentce interval.

Results were fully adjusted for age, sex, educational level, body mass index, season, year, physical labour at work, smoking, drinking, vegetable intake, fruit intake, occupational exposure to dust & organic solvent, hypertension, diabetes, hyperlipidemia, self-reported cardiovascular disease and self-reported cancer.

The 1^st^, 2^nd^, 3^rd^ and 4^th^ quartile of PM_2.5_ was <21.56, 21.56-24.03, 24.03-29.76 and ≥29.76 μg/m³ respectively.

| **Lung function** | **Inactive-PA** | | **Low-PA** | | **Moderate-PA** | | **High-PA** | |
| --- | --- | --- | --- | --- | --- | --- | --- | --- |
|  | **% Difference** | ***P*** | **% Difference** | ***P*** | **% Difference** | ***P*** | **% Difference** | ***P*** |
| **FVC** |  |  |  |  |  |  |  |  |
| 4th Quartile | Ref | - | 0.09 (-0.10, 0.28) | 0.335 | 0.55 (0.35, 0.75) | <0.001 | 1.13 (0.90, 1.36) | <0.001 |
| 3rd Quartile | 3.64 (3.43, 3.85) | <0.001 | 4.02 (3.80, 4.25) | <0.001 | 3.67 (3.43, 3.90) | <0.001 | 3.78 (3.53, 4.03) | <0.001 |
| 2nd Quartile | 4.39 (4.18, 4.60) | <0.001 | 4.33 (4.10, 4.56) | <0.001 | 4.71 (4.47, 4.95) | <0.001 | 5.39 (5.14, 5.65) | <0.001 |
| 1st Quartile | 6.23 (6.01, 6.45) | <0.001 | 5.59 (5.35, 5.84) | <0.001 | 6.23 (5.98, 6.47) | <0.001 | 6.94 (6.69, 7.20) | <0.001 |
| **FEV_1_** |  |  |  |  |  |  |  |  |
| 4th Quartile | Ref | - | 0.19 (0.18, 0.19) | <0.001 | 0.47 (0.46, 0.48) | <0.001 | 0.53 (0.52, 0.54) | <0.001 |
| 3rd Quartile | 3.91 (3.90, 3.92) | <0.001 | 4.00 (3.99, 4.01) | <0.001 | 4.25 (4.24, 4.26) | <0.001 | 4.40 (4.39, 4.41) | <0.001 |
| 2nd Quartile | 4.79 (4.78, 4.80) | <0.001 | 4.45 (4.44, 4.46) | <0.001 | 5.16 (5.15, 5.17) | <0.001 | 5.99 (5.98, 6.00) | <0.001 |
| 1st Quartile | 6.76 (6.75, 6.77) | <0.001 | 6.44 (6.43, 6.45) | <0.001 | 7.25 (7.24, 7.26) | <0.001 | 8.54 (8.53, 8.55) | <0.001 |
| **MMEF** |  |  |  |  |  |  |  |  |
| 4th Quartile | Ref | - | 0.47 (0.16, 0.77) | 0.003 | 0.11 (-0.21, 0.44) | 0.488 | -0.44 (-0.80, -0.07) | 0.019 |
| 3rd Quartile | 2.41 (2.09, 2.72) | <0.001 | 2.69 (2.34, 3.05) | <0.001 | 2.29 (1.93, 2.64) | <0.001 | 2.31 (1.92, 2.71) | <0.001 |
| 2nd Quartile | 4.24 (3.92, 4.57) | <0.001 | 4.04 (3.68, 4.40) | <0.001 | 4.34 (3.98, 4.71) | <0.001 | 5.23 (4.83, 5.62) | <0.001 |
| 1st Quartile | 7.85 (7.51, 8.20) | <0.001 | 6.86 (6.48, 7.24) | <0.001 | 8.27 (7.88, 8.67) | <0.001 | 10.64 (10.23, 11.05) | <0.001 |

**Table S6. Comparison on lung function of non-smokers with different levels of physical activity and PM_2.5_ exposures (N=423,491).**

Abbreviations: PA, physical activity; FVC, forced vital capacity; FEV_1_, forced expiratory volume in 1 second; MMEF, maximum mid-expiratory flow.

Lung function was logarithmically transformed to normalise the data for analysis and then the original scale was transformed back to present the effects as percentage (%) difference in lung function parameters with 95% confidentce interval.

Results were fully adjusted for age, sex, educational level, body mass index, season, year, physical labour at work, smoking, drinking, vegetable intake, fruit intake, occupational exposure to dust & organic solvent, hypertension, diabetes, hyperlipidemia, self-reported cardiovascular disease and self-reported cancer.

The 1^st^, 2^nd^, 3^rd^ and 4^th^ quartile of PM_2.5_ was <21.70, 21.70-24.17, 24.17-29.22 and ≥29.22 μg/m³ respectively.

| **Lung function** | **Inactive-PA** | | **Low-PA** | | **Moderate-PA** | | **High-PA** | |
| --- | --- | --- | --- | --- | --- | --- | --- | --- |
|  | **% Difference** | ***P*** | **% Difference** | ***P*** | **% Difference** | ***P*** | **% Difference** | ***P*** |
| **FVC** |  |  |  |  |  |  |  |  |
| 4th Quartile | Ref | - | 0.33 (0.13, 0.53) | 0.002 | 0.63 (0.41, 0.85) | <0.001 | 1.11 (0.86, 1.37) | <0.001 |
| 3rd Quartile | 2.99 (2.78, 3.21) | <0.001 | 3.37 (3.14, 3.61) | <0.001 | 3.20 (2.95, 3.45) | <0.001 | 3.34 (3.06, 3.61) | <0.001 |
| 2nd Quartile | 3.75 (3.53, 3.97) | <0.001 | 3.71 (3.47, 3.96) | <0.001 | 3.91 (3.66, 4.16) | <0.001 | 4.88 (4.61, 5.15) | <0.001 |
| 1st Quartile | 5.19 (4.96, 5.42) | <0.001 | 5.08 (4.83, 5.34) | <0.001 | 5.76 (5.50, 6.03) | <0.001 | 6.26 (5.99, 6.54) | <0.001 |
| **FEV_1_** |  |  |  |  |  |  |  |  |
| 4th Quartile | Ref | - | 0.13 (-0.07, 0.33) | 0.208 | 0.73 (0.51, 0.95) | <0.001 | 1.11 (0.85, 1.37) | <0.001 |
| 3rd Quartile | 3.57 (3.35, 3.79) | <0.001 | 3.77 (3.53, 4.01) | <0.001 | 3.71 (3.46, 3.96) | <0.001 | 3.66 (3.38, 3.93) | <0.001 |
| 2nd Quartile | 4.36 (4.14, 4.59) | <0.001 | 3.96 (3.72, 4.21) | <0.001 | 4.52 (4.27, 4.78) | <0.001 | 5.06 (4.79, 5.33) | <0.001 |
| 1st Quartile | 6.35 (6.11, 6.58) | <0.001 | 6.01 (5.75, 6.27) | <0.001 | 6.97 (6.70, 7.24) | <0.001 | 7.92 (7.64, 8.20) | <0.001 |
| **MMEF** |  |  |  |  |  |  |  |  |
| 4th Quartile | Ref | - | 0.47 (0.14, 0.80) | 0.005 | 0.19 (-0.16, 0.54) | 0.286 | -0.35 (-0.76, 0.05) | 0.090 |
| 3rd Quartile | 2.39 (2.06, 2.73) | <0.001 | 2.38 (2.01, 2.76) | <0.001 | 1.65 (1.27, 2.04) | <0.001 | 2.47 (2.04, 2.90) | <0.001 |
| 2nd Quartile | 3.85 (3.51, 4.20) | <0.001 | 3.90 (3.52, 4.28) | <0.001 | 4.22 (3.83, 4.62) | <0.001 | 5.29 (4.87, 5.72) | <0.001 |
| 1st Quartile | 7.83 (7.46, 8.20) | <0.001 | 6.91 (6.50, 7.32) | <0.001 | 8.08 (7.67, 8.50) | <0.001 | 10.22 (9.77, 10.67) | <0.001 |

**Table S7. Associations of lung function with habitual physical activity and PM_2.5_ exposure in Taiwanese adults (N=567,557)**

|  | **Adjusted Model 1 ^a^** | | **Adjusted Model 2 ^a^** | |
| --- | --- | --- | --- | --- |
|  | **% Difference** | ***P*** | **% Difference** | ***P*** |
| **FVC** |  |  |  |  |
| Low-PA | 0.12 (0.03, 0.20) | 0.006 | 0.01 (-0.08, 0.09) | 0.854 |
| Moderate-PA | 0.38 (0.29, 0.47) | <0.001 | 0.37 (0.28, 0.46) | <0.001 |
| High -PA | 0.89 (0.78, 1.00) | <0.001 | 0.92 (0.81, 1.03) | <0.001 |
| Per 10 MET-h | 0.24 (0.22, 0.27) | <0.001 | 0.22 (0.20, 0.25) | <0.001 |
| **PM_2.5_** |  |  |  |  |
| 2nd Quartile | -1.35 (-1.44, -1.25) | <0.001 | -1.48 (-1.57, -1.39) | <0.001 |
| 3rd Quartile | -2.00 (-2.11, -1.89) | <0.001 | -2.09 (-2.20, -1.98) | <0.001 |
| 4th Quartile | -4.11 (-4.25, -3.96) | <0.001 | -4.56 (-4.71, -4.42) | <0.001 |
| Per 10 μg/m^3^ | -2.40 (-2.48, -2.33) | <0.001 | -2.36 (-2.43, -2.28) | <0.001 |
|  |  |  |  |  |
| **FEV_1_** |  |  |  |  |
| Low-PA | -0.10 (-0.18, -0.02) | 0.019 | -0.14 (-0.22, -0.05) | 0.001 |
| Moderate-PA | 0.27 (0.18, 0.37) | <0.001 | 0.21 (0.12, 0.31) | <0.001 |
| High -PA | 0.95 (0.84, 1.06) | <0.001 | 0.92 (0.81, 1.03) | <0.001 |
| Per 10 MET-h | 0.24 (0.23, 0.25) | <0.001 | 0.24 (0.21, 0.26) | <0.001 |
| **PM_2.5_** |  |  |  |  |
| 2nd Quartile | -1.82 (-1.83, -1.81) | <0.001 | -1.68 (-1.77, -1.58) | <0.001 |
| 3rd Quartile | -2.55 (-2.56, -2.54) | <0.001 | -2.63 (-2.74, -2.52) | <0.001 |
| 4th Quartile | -5.87 (-5.88, -5.86) | <0.001 | -4.91 (-5.06, -4.76) | <0.001 |
| Per 10 μg/m^3^ | -2.85 (-2.86, -2.84) | <0.001 | -2.89 (-2.90, -2.88) | <0.001 |
|  |  |  |  |  |
| **MMEF** |  |  |  |  |
| Low-PA | -0.13 (-0.27, 0) | 0.056 | -0.20 (-0.33, -0.06) | 0.005 |
| Moderate-PA | 0.03 (-0.12, 0.18) | 0.703 | -0.10 (-0.25, 0.05) | 0.206 |
| High -PA | 0.68 (0.51, 0.86) | <0.001 | 0.80 (0.63, 0.98) | <0.001 |
| Per 10 MET-h | 0.22 (0.18, 0.26) | <0.001 | 0.23 (0.19, 0.27) | <0.001 |
| **PM_2.5_** |  |  |  |  |
| 2nd Quartile | -3.49 (-3.63, -3.34) | <0.001 | -3.24 (-3.39, -3.09) | <0.001 |
| 3rd Quartile | -5.17 (-5.34, -5.00) | <0.001 | -4.88 (-5.05, -4.71) | <0.001 |
| 4th Quartile | -6.50 (-6.72, -6.28) | <0.001 | -6.29 (-6.51, -6.07) | <0.001 |
| Per 10 μg/m^3^ | -3.19 (-3.30, -3.07) | <0.001 | -3.20 (-3.32, -3.09) | <0.001 |

Abbreviations: PA, physical activity; FVC, forced vital capacity; FEV_1_, forced expiratory volume in 1 second; MMEF, maximum mid-expiratory flow.

Lung function was logarithmically transformed to normalise the data for analysis and then the original scale was transformed back to present the effects as percentage (%) difference in lung function parameters with 95% confidence interval. The effects were presented as % difference in lung function with 95% confidence level.

Participants who were in inactive-PA category or in the 1^st^ quartile of PM_2.5_ comprised the reference group.

The 1^st^, 2^nd^, 3^rd^ and 4^th^ quartile of PM_2.5_ was <21.67, 21.67-24.14, 24.14-28.81 and ≥28.81 μg/m³, respectively.

^a^ Model 1: adjusted for age, sex, educational level, body mass index, season, year, physical labour at work, smoking, drinking, vegetable intake and fruit intake; Model 2 further adjusted for occupational exposure to dust & organic solvent.
